# Feasibility of investigating methylphenidate for the treatment of sarcoidosis-associated fatigue (the FaST-MP study): a double-blind, parallel-arm randomised feasibility trial

**DOI:** 10.1136/bmjresp-2020-000814

**Published:** 2021-05-21

**Authors:** Christopher Atkins, Andy Jones, Allan B Clark, Andrea Stockl, Richard Fordham, Andrew M Wilson

**Affiliations:** 1 Department of Respiratory Medicine, Norfolk and Norwich University Hospital NHS Trust, Norwich, UK; 2 Norwich Medical School, University of East Anglia, Norwich, UK

**Keywords:** sarcoidosis

## Abstract

**Introduction:**

Sarcoidosis-associated fatigue (SAF) is a common clinical problem with limited treatment options. This study was undertaken to determine the feasibility of performing a definitive trial to determine the clinical efficacy methylphenidate in SAF.

**Methods:**

This was a parallel-arm, double-blind, placebo-controlled randomised controlled feasibility trial enrolling sarcoidosis patients reporting significant fatigue. Patients with a Fatigue Assessment Scale score of more than 21 were randomised to receive up to either 10 mg two times per day methylphenidate or identical placebo capsules two times per day, in a dose escalation fashion, for up to 24 weeks. Outcomes included number of participants eligible and willing to participate, withdrawal rates, adherence rates and ability to maintain blinding.

**Results:**

Of 385 patients screened, 56 (14.5%) were eligible and 23 (41% of eligible patients) were randomised. No withdrawals occurred. One participant in the methylphenidate arm discontinued study medications due to chest pain. The side effect profile was not different between the groups. Median medication adherence rates were 98% and 99% in the methylphenidate and placebo arms, respectively. A greater proportion of participants receiving methylphenidate predicted their allocated treatment while blinded compared with those receiving placebo (93.3% vs 57.1%). The investigator could not predict the treatment allocation. Both groups showed clinically meaningful improvements in fatigue from baseline, although no between-group difference was seen.

**Conclusions:**

The data support the feasibility of performing a double-blind parallel trial powered to determine the clinical efficacy of methylphenidate for SAF, however, a multicentre study will be required.

**Trial registration number:**

NCT02643732.

**Key messages**

- Is it feasible to attempt a full-size randomised controlled trial of neurostimulants for sarcoidosis-associated fatigue and what should that trial look like?
- Methylphenidate appeared safe and well tolerated overall. Recruitment was such that a multi-centre study is required and would be improved by some adjustments to study design and follow-up.
- The FaST-MP study provides important data when considering future randomised trials for sarcoid-associated fatigue, as well as suggesting that methylphenidate treatment is acceptable.

## Introduction

Sarcoidosis is frequently complicated by constitutional symptoms including fatigue,^1^ which can be chronic and difficult to manage,^2^ significantly impairing quality of life.^1^ While several treatments have been investigated,^3^ many are systemic immunosuppressant therapies associated with significant side effects or costs, and may not be appropriate in cases where sarcoidosis-associated fatigue (SAF) is the sole clinical manifestation.

Methylphenidate and its d-isomer dexmethylphenidate are piperidine-class stimulants which amplify dopaminergic neurotransmission in the basal ganglia.^4^ These drugs have been trialled for fatigue in other conditions, although the evidence for clinical efficacy has been mixed. In a placebo-controlled, double-blind trial in postchemotherapy patients with fatigue, dexmethylphenidate exhibited a clinically significant reduction in fatigue.^5^ A Cochrane review of treatments for cancer-related fatigue from five randomised controlled trials concluded that ‘the current evidence supports the use of psychostimulants in cancer-related fatigue’.^6^ A trial of methylphenidate in 109 HIV positive patients improved fatigue, with 41% of participants who received the drug demonstrating a greater than 50% improvement in Visual Analogue Scale (VAS) fatigue scores over a 6-week period.^7^ In contrast, no difference between methylphenidate and placebo over a 12-week period was seen in a cohort of 68 fatigued patients who had received radiotherapy for brain tumours.^8^ In chronic fatigue syndrome, a cross-over study of 60 patients found that only 17% reported improvements in fatigue scores over a 4-week duration.^9^

In SAF, the d-isomer of methylphenidate (dexmethylphenidate) has been trialled in a small cross-over study involving 10 patients and showed evidence of reduced fatigue over an 8-week period.^5^ However, questions remain regarding the feasibility of performing an appropriately powered trial to determine the clinical efficacy of methylphenidate for SAF. The proportion of patients with sarcoidosis eligible for such a trial is unknown. Sustainability of treatment effect beyond 8 weeks is unknown. Furthermore, the use of a cross-over design, used in previous studies investigating neurostimulants for SAF,^5^ has been suggested as inappropriate for these medications due to the risk of unblinding due to apparent treatment effects.^10^ This may lead to an increased observed effect size for stimulant medications, as shown in cross-over studies investigating their use in other conditions.^11^

The objective of the *Fatigue and Sarcoidosis: Treatment with Methylphenidate* (FaST-MP study was to determine the feasibility of performing a large-scale trial of methylphenidate for treatment of SAF. Clinical data were collected and analysed but the study was not powered to establish treatment effect.

## Methods

### Study design and setting

The full study protocol has been previously published.^12^ This was a parallel-arm, randomised, double-blind, placebo-controlled feasibility trial with participants allocated to methylphenidate or matched placebo on a 3:2 ratio. Participants were identified by screening the medical notes for reference to fatigue, including synonyms, in patients with sarcoidosis under active follow-up by the respiratory clinic at the Norfolk and Norwich University Hospital (NNUH), Norwich, UK or identified at participant identification centres (PICs) in East Anglia and referred to the trial team at NNUH. Potential participants were sent written trial information and then contacted by telephone; they were invited to a screening visit and consented by a trial physician. The trial was supported by the Norwich Clinical Trials Unit (NCTU) based at the University of East Anglia (UEA).

Participants received methylphenidate or an identical placebo for up to 24 weeks. Measurements of safety and efficacy were performed throughout the study and 6 weeks after completing study medications. After completing study medications but prior to study unblinding participants were offered the opportunity to participate in moderated focus groups to discuss their experience of the study. A protocol amendment was approved in April 2017 to permit truncation of follow-up for participants enrolled after December 2017.

### Eligibility

Patients were eligible if they had a diagnosis of sarcoidosis, stable disease and significant fatigue (Fatigue Assessment Scale (FAS) score of greater than 21 points on two occasions 2 weeks apart prior to starting medication, the average value used as baseline). Patients were excluded if they had an alternative cause for fatigue, including anaemia, hypercalcaemia, thyroid dysfunction or obstructive sleep apnoea (OSA). A full blood count, thyroid screen and electrolytes including calcium were measured at the screening visit. All patients were screened for symptoms of OSA using the STOP-Bang questionnaire^13^; participants scoring 4 or above, or who had symptoms suggestive of OSA irrespective of the STOP-Bang score, underwent overnight oximetry prior to inclusion to exclude OSA. Participants were also excluded if they were receiving medication known to interact with methylphenidate, or had risk factors for adverse events (AEs) including previous cardiovascular events, seizures, thyroid disorders, glaucoma or established liver disease. The full list of exclusion criteria is available.^12^

The study was registered on ClinicalTrials.gov (NCT02643732).

### Intervention and follow-up

The interventional drug was methylphenidate hydrochloride (Tranquilyn), overencapsulated with a gel capsule (Guys and St Thomas’ Pharmacy Manufacturing Unit, London, UK); the comparator was an identical placebo capsule. The initial dose was 10 mg two times per day of methylphenidate or one identical placebo capsule twice daily, increasing to 20 mg of methylphenidate (as 2×10 mg) two times per day or two identical placebo capsules twice daily after review at week 2, if appropriate.

Following their screening visit, eligible participants attended seven face to face study visits over a 24-week period (weeks 0, 2, 4, 6, 12, 18 and 24). Between study visits, participants were contacted by the study team via phone at weeks 1, 3, 5, 8, 10, 14, 16, 20 and 22 to review any potential side effects or safety concerns. Follow-up was truncated for participants enrolled after December 2017, who received methylphenidate for a minimum of 12 weeks.

### Randomisation and data collection

Randomisation was performed using block randomisation with blocks of five, in a 3:2 ratio favouring methylphenidate. Stratification was performed for baseline fatigue severity (FAS score 22–34 and 35–50). The randomisation sequence was produced by the study statistician, with allocation performed by the trial physician using a web-based data management system.

### Patient involvement

The FaST-MP study involved patients from conception through to completion. Patients with SAF were involved in the original application for funding and drafting of the original protocol. There was patient involvement in trial oversight through membership of the trial steering committee. Patients and trial participants were involved in reviewing of the final results following study completion.

### Sample size

A maximum sample of 30 participants was chosen in line with recommendations for sample sizes in feasibility studies.^14 15^

### Outcome measures

The primary feasibility outcomes of interest were:

1. Proportion of patients eligible for trial participation and willing to participate.
2. Recruitment rate and retention.
3. Number and type of AEs.
4. Indication of continuation of effect at stable dose during treatment period.
5. Ability to maintain blinding to allocation.
6. Number of missed or unfilled assessments.
7. Number of patients correctly using accelerometers.
8. Acceptability of study visits and assessments.
9. Overall perception of trial involvement.

Outcomes 1–7 were measured from quantitative data collected during the study. Outcomes 8 and 9 were assessed by analysis of the focus group discussion data.

Data were collected on clinical outcomes for exploratory analysis. Fatigue was measured using *FAS*^16^ and Functional Assessment of Chronic Illness Therapy*-*Fatigue *(*FACIT-Fatigue).^17^ FAS is a 10-point questionnaire ranging between 5 and 50 points with higher scores representing worse fatigue.^16^ The minimal clinically important difference (MCID) is four points.^18^ FACIT-Fatigue contains 13 items, with score ranging between 0 and 52 points and lower scores indicate worse fatigue.^19^ The MCID has been estimated to be between 3 and 6 points.^20^ Anxiety and depressive symptoms were assessed with the Hospital Anxiety and Depression Scale (HADS),^21^ with anxiety (HADS-A) and depression (HADS-D) scores reported separately. HADS is a 14-item questionnaire, of which seven questions are scored each for anxiety and depression. Each item is scored out of three, with a maximum and minimum score of 0 and 21 points, respectively, for both HADS-A and HADS-D. A score of over 10 points is considered to indicate significant anxiety or depressive symptoms. Additional questionnaires were also administered regarding quality of life (Kings Sarcoidosis Questionnaire), heath utility (EuroQoL-5 Dimension and Short Form-36), sleep quality (Pittsburgh Sleep Quality Index) and health costings, as well as spirometry. Though the results are not reported here, the rate of completion of these questionnaires by participants is reported in the results section.

Exercise capacity was measured using the modified incremental shuttle walk test (MSWT)^22^; this allows those with minimal cardiopulmonary impairment to be adequately stressed and has been shown to correlate with peak VO_2_ levels when compared with cardiopulmonary exercise testing in patients with sarcoidosis.^23^ Physical activity levels were captured by wrist-worn activity monitors (GENEActiv original, ActivInsight; Cambridgeshire, UK). The feasibility of repeatedly using these devices was determined by evaluating the number of devices safely returned with ‘valid’ data (at least 10 hours wear period for at least 2 weekdays and 2 weekend days).^24^ Assessments of blood pressure and pulse, weight, biochemistry (full blood count, urea and electrolytes and liver function), ECG and AEs occurred at each visit.

At the final study visit (week 24, or week 12 or 18 for patients recruited after December 2017) participants completed an exit questionnaire. This asked if the participant would wish to continue the medication if the option were available, if they found participation in the study useful and if they would take part in the study given the chance again. It also asked the participant to predict whether they had been receiving methylphenidate or placebo; the investigator separately completed their prediction of the participant’s allocation.

All participants were invited to attend face-to-face moderated, audiorecorded focus groups to discuss experiences during the trial, after they had completed medications but prior to unblinding. Three focus groups were undertaken, each containing between four and six participants. All focus groups were undertaken at the same location (UEA, Norwich)). A prespecified topic guide was used to facilitate discussion using open-ended questions. The two key topics investigated were the participants’ experience of trial participation (positive and negative), and invited suggestions for any changes to the study which might improve the future recruitment or retention of participants in any follow-up study to FaST-MP. The full methods for the focus groups are contained in online supplemental file S1.

10.1136/bmjresp-2020-000814.supp1 Supplementary data

### Statistical analysis

Feasibility and safety outcomes were reported as event rates. An exploratory analysis of the clinical data was performed on an intention to treat basis, including all participants who received study medications at any point. Plots were constructed for each outcome, displaying mean scores by allocation group with 95% CIs. Mean differences between allocation group were compared using a two-sample t-test (unadjusted analysis), with adjusted analysis of the data using a general linear regression model controlling for baseline values and initial fatigue severity. Continuation of effect was assessed using longitudinal measurements of FAS and FACIT-Fatigue. Any data not displayed or reported within this paper are included in online supplemental file S2. Analysis was performed using Stata statistical software V.14 (StataCorp).

10.1136/bmjresp-2020-000814.supp2 Supplementary data

### Blinding

Participants, care providers and investigators were blinded to allocation; the placebo and active treatments appeared identical and were dispensed in identical containers. Trial pharmacists at the NNUH could identify allocation due to unequal arm size. Pharmacy monitoring was performed by an independent member of NCTU to ensure unblinding did not occur. Unblinding occurred only after all data had been collected.

## Results

### Screening and recruitment

**Figure 1:**
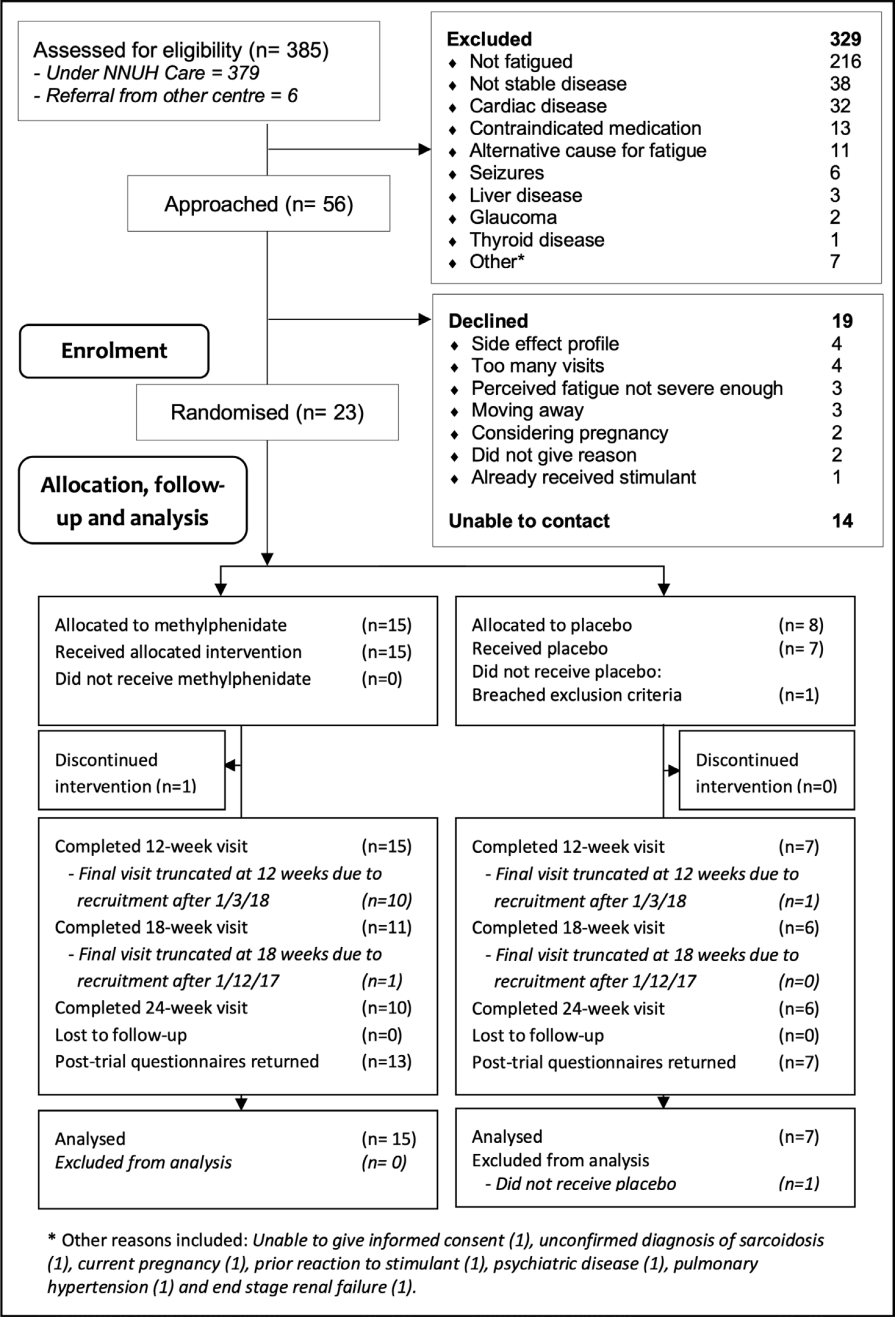
Recruitment and screening (CONSORT statement). CONSORT, Consolidated Standards of Reporting Trials; NNUH, Consolidated Standards of Reporting Trials.

Recruitment occurred between 11 July 2016 and 3 February 2018; the trial ended because of a prespecified end date. In total 385 patients were screened, of which 379 were under the care of NNUH and six referred from PIC sites. Fifty-six patients (14.5%) were potentially eligible, of which twenty-three participants agreed to participate (41.1% of all eligible patients, 6.0% of all screened patients). At NNUH alone, 52 eligible patients were identified (13.7% of NNUH patients); of those, 19 patients (36.5% of eligible NNUH patients, 5.0% of all NNUH patients) agreed to participate. Twenty-two participants received their allocated intervention; one participant was excluded after randomisation but prior to receiving their allocated intervention due to identification of an exclusion factor. Recruitment averaged 1.4 participants/month overall (1.2 from NNUH). No participants withdrew from the study. Figure 1 shows screening, trial recruitment and flow.

Baseline characteristics of participants who received their allocated intervention are shown in table 1.

**Table 1:** Baseline characteristics by group

Variable	Methylphenidate (n=15)	Placebo (n=7)
Age at randomisation (years)	55.5 (10.1)	55.4 (7.7)
Gender (%)		
Male	10 (66.7)	3 (42.9)
Female	5 (33.3)	4 (57.1)
Smoking status (%)		
Current	0 (0.0)	0 (0.0)
Ex	4 (26.7)	3 (42.9)
Never	11 (73.3)	4 (57.1)
Alcohol intake (units/week)	5.3 (7.6)	4.7 (10.3)
Body mass index (kg/m^2^)	30.3 (4.5)	33.8 (7.6)
Disease duration at randomisation (years)	6.7 (7.1)	6.0 (7.8)
>3 years	9 (60.0)	4 (57.1)
1–3 years	2 (13.3)	2 (28.6)
<1 year	4 (26.7)	1 (14.3)
Pulmonary disease (%)	15 (100.0)	7 (100.0)
Extrapulmonary disease (%)	9 (60.0)	3 (42.9)
Ethnicity		
Caucasian	15 (100.0)	7 (100.0)
Current treatment for sarcoidosis	4 (26.7)	4 (57.2)
Prednisolone	3 (20.0)	1 (14.3)
Methotrexate	1 (6.7)	2 (28.6)
Azathioprine	0 (0.0)	1 (14.3)
Baseline FAS Score	35.9 (7.7)	35.9 (8.8)
FAS score 21–34 (%)	7 (46.7)	3 (42.9)
FAS score 34–50 (%)	8 (53.3)	4 (57.1)

Values presented as means (SD) or frequencies (%).

FAS, Fatigue Assessment Scale.

### Adherence and safety

Median adherence was 98% in the methylphenidate arm and 99% in the placebo arm. Only one participant had less than 80% adherence. Adherence rates did not change as the study progressed.

At the end of the study five participants in the methylphenidate arm were receiving a dose of 10 mg (one capsule) two times per day, nine participants were receiving 20 mg (two capsules) two times per day and one participant had discontinued the study medication (due to chest pains). All participants in the placebo arm were receiving two capsules twice daily at the end of the study

Ninety-six AEs were observed, including one serious event which occurred in the methylphenidate arm; this was attributed to a concurrent medication. The number of participants developing at least one AE in each organ system is shown in table 2. No cardiac events, ECG abnormalities or biochemical abnormalities requiring discontinuation occurred in any participant. Participants receiving methylphenidate had a weight reduction of 2.9 kg; no weight change was observed in the placebo arm.

**Table 2:** Adverse event (AE) rates by treatment allocation; number of participants in each arm developing at least one AE within each individual organ system

CTCAE system class	Methylphenidate No of participants with ≥1 event (%)	Placebo No of participants with ≥1 event (%)
Ear and labyrinth	2 (13.3)	0
Eye	1 (6.7)	3 (42.9)
Gastrointestinal	7 (46.7)	1 (14.3)
General disorders	2 (13.3)	2 (28.6)
Infections and infestations	1 (6.7)	1 (14.3)
Investigations	2 (13.3)	0
Metabolism and nutrition	1 (6.7)	0
Musculoskeletal	5 (33.3)	1 (14.3)
Nervous system	10 (66.7), 1 SAE (6.7)	3 (42.9)
Psychiatric	5 (33.3)	3 (42.9)
Respiratory	7 (46.7)	6 (85.7)
Reproductive system and breast	1 (6.7)	0
Skin and subcutaneous tissue	4 (26.7)	1 (14.3)
Vascular disorders	2 (13.3)	0
Any	14 (93.3)	7 (100.0)

CTCAE, Common Terminology Criteria for Adverse Events; SAE, serious AE.

### Data completeness

The proportion of missing data points was 5.0% or less for all outcomes except the MSWT (11.7% data points missing), due to temporary lack of access to suitable facilities (table 3). Activity monitors were worn reliably. Of 60 wear periods, 59 (98.3) had devices returned safely of which 54 (90.0%) contained valid data.

**Table 3:** Completion rates for questionnaires and other outcomes performed during the study

Outcome	Expected data points—n	Missing data points—n (%)
FAS	165	2 (1.2)
FACIT-Fatigue	165	2 (1.2)
HADS	121	4 (3.3)
KSQ	121	3 (2.5)
EQ5D	121	3 (2.5)
SF36	121	4 (3.3)
Safety*	104	5 (4.8)
PSQI†	43	2 (4.7)
Spirometry (FEV1 and FVC)‡	60	3 (5.0)
MSWT§	60	7 (11.7)
Activity monitor data¶	60	3 (5.0)
Total	1142	30 (2.6)

*Safety questionnaire was administered up to week 12; participants completing a truncated time period who completed study medications at week 12 did not all receive safety questionnaires at their final visit (4 out of 5 missing data points).

†PSQI only administered following major amendment approved in April 2017; expected data points refers to the number of visits where the questionnaire should have been administered after the study amendment was approved.

‡All missing spirometry values occurred in a single participant who was unable to perform the test without suffering syncope.

§Six of the seven missing MSWT values occurred due to loss of facilities to undertake the test.

¶Missing data points for activity watches refers to an unreturned device (one missing data point) or device not worn during wear period (two missing data points).

EQ5D, EuroQoL 5 Dimension 5 Level scale; FACIT-Fatigue, Functional Assessment of Chronic Illness Therapy-Fatigue; FAS, Fatigue Assessment Scale; FEV1, forced expiratory volume in 1s; HADS, Hospital Anxiety and Depression Scale; KSQ, Kings Sarcoidosis Questionnaire; MSWT, modified shuttle walk test; PSQI, Pittsburgh Sleep Quality Index; SF36, Short Form 36.

### Exploratory clinical efficacy

**Figure 2:**
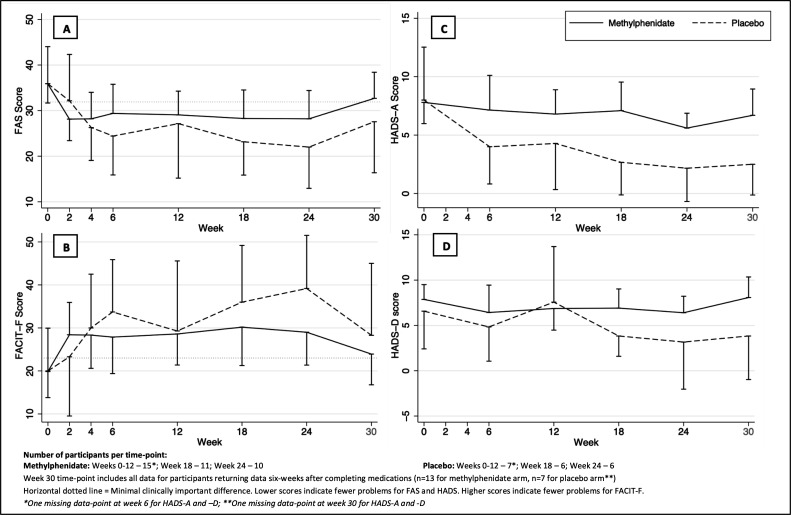
Change in Fatigue Assessment Scale (FAS) (A), Functional Assessment of Chronic Illness Therapy-Fatigue (FACIT-F) (B), Hospital Anxiety and Depression Scale-Anxiety (HADS-A) (C) and Hospital Anxiety and Depression Scale-Depression (HADS-D) (D) scores from baseline values over time, presented by allocation. Results are mean values with 95% CIs.

Baseline mean FAS scores were 35.9 in both arms (SD 7.8). Baseline FACIT-Fatigue score was 19.9 in the methylphenidate arm and 20.0 in the placebo arm. Changes in fatigue scores were similar in both arms (figure 2). At week 12 and 24 a similar proportion of participants in each arm met the MCID for the FAS score (73.3% and 80.0% in the methylphenidate arm at weeks 12 and 24, respectively, 71.4% and 83.3% in the placebo arm). Both groups showed an increase in fatigue 6 weeks postmedication.

Mean baseline HADS-A and HADS-D scores were 7.8 and 7.9, respectively, for the methylphenidate group, 8.0 and 6.6 respectively for the placebo group. Mean HADS-A score was 2.5–4.4 points lower in the placebo arm than the methylphenidate arm across the medication period. HADS-D scores remained similar throughout the study. Figure 2 shows the change in HADS scores over time in each group.

### Post-trial outcomes

Nineteen participants (12 methylphenidate, 7 placebo) completed the exit questionnaire. A greater proportion of participants in the methylphenidate arm wanted to continue the drug (91.7%) compared with placebo (71.4%). All participants stated that they found participation in the trial useful and would participate if given the opportunity again. Blinding was maintained in the placebo arm but participants receiving methylphenidate were aware when they received the medication; 14 of the methylphenidate group correctly predicted their allocation (93.3%), compared with four receiving placebo (57.1%). The investigator predicted allocation to methylphenidate less accurately, correctly predicting the allocation of 11 participants in the methylphenidate arm (73.3%, but was slightly better in predicting allocation to placebo (five participants, 71.4%).

Fourteen participants attended post-trial focus groups. Participants talked positively about the study and the impact of treatment on their lives; they were keen to see methylphenidate available as an option for SAF. The number of questionnaires was considered an issue, particularly as there was overlap between some questionnaires. It was suggested that the fatigue outcome measures were ‘vague’ and might miss improvements that participants felt were important to them, with participants in one of the focus groups suggesting simpler but more frequent measures of fatigue, such as a VAS, might be used alongside a formal questionnaire such as FAS. Alternatively, a self-report diary was suggested. The full outcomes from the focus groups are included in online supplemental file S1.

## Discussion

The FaST-MP study showed a phase-III trial is feasible but a multicentre study is required and modifications from this trial design will improve deliverability. The trial did not meet its recruitment target, partly due to the cautious entry criteria and intensive visit schedule, although retention of participants and adherence to the study measurements was excellent. Methylphenidate was safe and well tolerated. Our experience with wrist-worn activity monitors suggests that the using these devices is feasible and carries advantages over formal measures of exercise capacity such as MSWT. The use of the FAS questionnaire should be the primary outcome for any future trial, but could be complemented by a VAS, with an outcome assessment at 3 months. We have shown that a parallel study is appropriate but a cross-over design runs the risk of unblinding.

The FaST-MP study had several strengths. This was a mixed-methods study which carefully evaluated safety of patients with sarcoidosis receiving methylphenidate. Through screening a large number of patients with pulmonary sarcoidosis under active follow-up by a large regional hospital it identified the proportion of patients potentially eligible to undertake a future study, excluding patients with alternative causes of fatigue. Screening for fatigue was robust, including measuring fatigue with a validated score on two separate occasions, to ensure participants’ fatigue was both significant and stable. It used numerous end-points. Using wrist-worn activity monitors to monitor change in daily activity was successful and could be replicated in future studies.

There were limitations. The recruitment target was not met. The frequent visit schedule deterred some patients and the entry criteria excluded a high proportion of patients for reasons of safety. As a single centre, our recruitment and retention rates may be better than those seen in a multicentre study. While the proportion of patients screened reporting fatigue was lower some previous studies,^25^ it is in line with UK data from the BTS sarcoidosis registry.^26^ This may be because in this study fatigue was reported by physicians (in clinic correspondence), whereas data from other studies was reported by patients; the prevalence of fatigue in this study may be an underestimate. This may mean some patients with fatigue were missed. Although the trial information was prominently displayed to patients attending the respiratory outpatient department, thereby allowing self-referral to the research team, this may still have meant that patients not reporting fatigue were not offered the chance to participate. Offering all patients with sarcoidosis the opportunity to participate and then screening them with the FAS questionnaire may have identified additional patients with an FAS score >21 points, although these patients may not have felt fatigue was a significant problem and therefore would not be candidates for neurostimulants on a clinical basis.

The FAS instrument remains the outcome of choice for measuring fatigue; it has been recommended for any study measuring fatigue in patients with sarcoidosis.^27^ It is validated in sarcoidosis,^16^ has a known MCID,^18^ and is widely used.^1^ FAS was reliably completed in this study. The changes seen in FAS scores were mirrored by FACIT-Fatigue, so an alternative fatigue measure added little. Focus group discussions suggested that FAS may miss changes in fatigue important to individuals. One change suggested by focus group participants was the addition of a simple fatigue VAS, which could be used alongside FAS, but potentially administered more frequently. While fatigue VAS instruments have been used in other conditions^28^ they have not been evaluated in sarcoidosis. Therefore, while they may provide a useful adjunct to the FAS score, they should not replace it in future trials.

The experience with wrist-worn activity monitors suggests these devices are feasible to use within a subsequent trial; the proportion of patients returning at least minimum valid data was high and comparable to observational studies using posted accelerometer devices such as UK Biobank.^29^ We were able to reliably collect valid data across the trial with only one device lost. The use of these devices is preferable to exercise tests due to difficulties with reliably accessing suitable facilities at multiple sites. In this study we encountered problems securing space for the MSWT which requires a 10 m track; activity monitors do not need this requirement. Previous data has also suggested a weak correlation between exercise capacity (measured by a 6 min walk test) and fatigue.^30^ By measuring activity in free-living conditions, activity monitors provide different information directly linking to daily exercise levels. This may be preferable as previous data has suggested an association between fatigue and the number of bouts of physical activity.^31^

Given that the planned recruitment was not met, consideration must be given to ways of increasing recruitment. Over 20% of potentially eligible participants declined to participate due to the visit schedule. Reducing the number of study visits may have encouraged a greater number of patients to participate. The safety profile of methylphenidate in this study suggests that fewer safety visits are required. No issues with blood pressure or pulse were identified during the study and screening for AEs could be done via phone or remote monitoring. Methylphenidate is considered safe in other conditions including in children and adults,^32^ in those with attention deficit hyperactivity disorder^33^ and those with Alzheimer’s disease.^34^ Furthermore, the exclusion criteria for FaST-MP was deliberately risk-averse. These restrictions may be relaxed. For example, a number of screened patients (2.5%) were excluded due to the use of tricyclic antidepressants; these drugs need not be a strict exclusion criterion but could be continued with monitoring.

We questioned whether it was possible to undertake a future blinded study of neurostimulants in SAF, given the previous concerns with cross-over studies.^10^ Most patients receiving methylphenidate correctly predicted their allocation; neither those in the placebo arm nor the investigators were reliably able to. Difficulty maintaining blinding in the methylphenidate group suggests it would be challenging to maintain blinding in a cross-over study, as previously noted.^10^ In any future study, it is important that a formal assessment of blinding efficacy be performed to ensure that blinding has not inadvertently been broken. There are a number of methods for doing this, which can easily be added to the questionnaires delivered during the study and would ensure robustness of the outcomes. These include the James’ Blinding Index (BI) and Bang BI, which require participants to express their prediction regarding allocation and the degree of certainty with which they make the prediction; these predictions can then be statistically compared between groups to determine if blinding has been maintained.^35^

Though participants in both arms showed reduced fatigue, the study was not powered for clinical effect. The small sample size makes it difficult to draw conclusions about the performance of either arm, though both subjective and objective measures have been seen to improve in the placebo arms of trials in other conditions.^36^ We performed multiple baseline fatigue measurements prior to commencing medications given the subjective nature of fatigue, in line with previous suggestions,^36^ though other aspects of the trial design may have influenced the outcomes in the placebo arm. The high level of contact with the study team may have reduced anxiety levels^37 38^; anxiety is known to moderate fatigue.^39^ Furthermore, because of the small trial team, participants met the same investigator and support team which may have increased this effect. Participants may have subconsciously filled a ‘good participant’ role, striving to meet the study hypothesis.^40^ Another impact of the high level of contact with the trial team is potentially the Hawthorne effect, or ‘research participant effect’,^41^ where the persistent interaction and completion of study activities can alter perception of symptoms. Overall, the level of interaction with the trial team meant that the placebo arm did not represent usual care. A future trial would ideally have less interaction between the trial team and participants, or would vary the investigator meeting participants.

We have shown that a multicentre trial of methlyphenidate for SAF is feasible but modification of the design is required to improve delivery; intensive safety monitoring is not required. A parallel-arm design is appropriate whereas a cross-over study would introduce unblinding. A definitive trial can now be considered especially given the lack of treatments for this common symptom of sarcoidosis.

## Data Availability

The dataset from this trial is available from the corresponding author on reasonable request.
