# Supplementary material for: Feasibility of investigating methylphenidate for the treatment of sarcoidosis-associated fatigue (the FaST-MP study): a double-blind, parallel-arm randomised feasibility trial
Source: BMJ Open Respir Res. 2021 May 21;8(1):e000814. doi: 10.1136/bmjresp-2020-000814 (PMC8144057; doi:10.1136/bmjresp-2020-000814)
Supplement: Supplementary data [file bmjresp-2020-000814supp001.pdf]

Supplementary File S1 – Focus group methods and outcomes relating to trial design and performance

Methods:

Three audio-recorded, moderated focus groups were planned for participants of the study who had completed their trial participation (i.e. after their post-trial/week 30 questionnaires had been returned). All focus groups occurred prior to study unblinding, therefore all participants were blinded to their allocation.

Each focus-group was to contain between four and six participants (minimum group size of four participants), dependent upon participant availability. All focus groups were undertaken at the same location (University of East Anglia). A pre-specified topic guide was used to facilitate discussion using open-ended questions (the topic guide can be found at the end of this document). The two key topics being investigated were the participants' experience of trial participation (both positive and negative) and whether they could suggest any changes to the study which might improve the future recruitment or retention of participants in any follow-up study to FaST-MP.

Each topic heading included an initial series of open-ended questions, some containing prompts to aid discussion should this be required. To avoid embarrassment (in the event of discussing sensitive issues relating to impact of fatigue), questions pertaining to the impact of fatigue were not included in the topic guide. Where participants began to discuss the impact of sarcoidosis-associated fatigue on their lives during the course of discussion within the focus groups, these conversations were allowed to continue and were permitted as long as participants were comfortable continuing the discussion. No additional questions exploring the impact of sarcoidosis-associated fatigue was asked by the moderator during such discussions.

All participants within the focus group completed written informed consent as part of the consent process; this was not a mandatory aspect of the study and only those providing written consent were approached about participating in the focus groups.

Audio data collected from the focus-groups was transcribed, with transcription and participant identity within the transcripts checked and confirmed by the lead researcher (CA). The audio data was complemented by written notes collected by the moderator present within the discussions. A research assistant was also present during each focus group to assist with the running of the session.

Analysis of the data was undertaken using a thematic analysis approach, according to previous recommendations by Braun and Clarke(1). This involved the following steps:

1. Familiarisation with the data
2. Generating initial codes
3. Searching for themes
4. Reviewing themes
5. Defining and naming themes
6. Report production

The lead researcher (CA) undertook data familiarisation, code generation and identification of initial themes. These initial themes were then modified through back-and-forth discussions between the lead researcher (CA) and a member of the trial team with specialism in this area (AS), who was the supervisor for the qualitative aspect of this project.

Coding of themes was assisted by NVIVO 11 software.

Objectives

The focus groups were performed to explore positive and negative aspects of participation within the FaST-MP study, including possible improvements or changes for any follow-on study from FaST-MP which may improve participant recruitment, retention or experience within that study.

Results

Fourteen participants from the *FaST-MP* study took part in this study across the three focus groups that were undertaken; this represents 63.6% of the total number of participants within the *FaST-MP* study. Each focus group took between 75 and 120 minutes in total. The age, gender and allocation to treatment or placebo arm within the *FaST-MP* study are shown in table 1 below. The focus groups took place before unblinding of the *FaST-MP* study, therefore none of the participants were aware of their treatment allocation at the time of the focus groups.

Table 1 – Focus group participant demographics

**Focus group number**	**ID Number**	**Age**	**Gender**	**Baseline Fatigue Score *(FAS)***	**Allocation arm in *FaST-MP***
**1**	002	59	F	34	Methylphenidate
	003	71	M	23	Methylphenidate
	005	53	F	30	Placebo
	006	56	M	26	Methylphenidate
**2**	001	69	F	26	Methylphenidate
	004	59	M	28	Placebo
	007	44	F	42	Placebo
	008	53	M	38	Methylphenidate
	009	52	M	22	Methylphenidate
**3**	011	65	M	40	Placebo
	013	65	F	45	Methylphenidate
	014	67	F	35	Placebo
	016	34	M	44	Methylphenidate
	019	69	M	31	Methylphenidate

Feedback on study design and outcome measures

Beyond the potential negative connotations and moral ambivalence of receiving stimulant medications for sarcoidosis-associated fatigue, specific negative aspects of the *FaST-MP* study design were raised by participants. One major problem related to a key aspect of the study, notably the questionnaires used for clinical outcome measures. For some of the participants, frustration was expressed at the number of questionnaires and the overlap between them. Although part of this was frustration at the tie it took, in some cases the symptoms of fatigue led to actual difficulty in completing the questionnaire pack, one participant recalled thinking "Gosh, how am I going to get through this [questionnaire pack]?" (013, Focus group 3) once she had discontinued the medications. Five of the participants felt that too many questionnaires were included, although two participants were happy with the number of questionnaires used if it meant that the study was not compromised for outcomes.

Whilst the number of questionnaires was considered a problem, a bigger issue was raised concerning the sensitivity of the questionnaires for collecting data which reflects improvements for the patients. The questionnaires were described as "not relevant," or "too vague" to adequately pick up the impact of fatigue on daily life; this lack of precision was echoed by others, especially relating to the variability of fatigue day-to-day and the

difficulty in giving an answer reflecting the severity of fatigue over a period of several weeks:

"I think the answers were very vague as well, especially the multiple-choice ones where it's 'sometimes' or 'often'. Well, and then it's over the last two weeks. Well, actually, I've had some good days and I've had some bad days but I can't write, "Well, actually, some of it was this, but some of it was this," or, like you said, it's often or sometimes." (016, Focus group 3)

An initial concern when designing the trial was that questionnaires were being administered too frequently, as often as every two weeks during the first six weeks of the study, but the feedback from focus group participants was that the visit schedule was not too onerous and yet the ability to track fatigue levels and the effect of medications was still hindered by not being able to measure fatigue levels frequently enough to obtain the resolution of data to adequately show change in fatigue. Clearly seeing or contacting a patient on a daily basis to score their fatigue levels is likely to be impractical and, for the participants, potentially tiresome. Participant 16, who had identified the vagueness in our method of measuring and tracking fatigue, offered a suggestion:

"I think, almost, like a daily diary. Not like a full questionnaire every day, but even if it's just notes for us. Some way of logging, "Today was a rubbish day, it took me almost two hours to get out of bed and I went back to bed because I was just too tired," and other days, "I got up, actually, I was really good until lunch time." (016, Focus group 3)

Whilst this form of data would allow greater insight into the impact of fatigue, it would present difficulties in quantitative analysis to detect change over time or change between groups. The use of technology was suggested, using a smartphone-type device as a way of administering a simple likert or visual analogue scale question on a daily basis relating to fatigue, with the option to add additional data as a diary entry if needed. This was considered interesting by the members of focus group 3, where this discussion had occurred, and offered a solution to what was considered a significant problem with the chosen outcome measures.

Another outcome measure being evaluated in the *FaST-MP* study was the use of activity monitors, worn for three seven-day periods during the study. The output from the devices, as shown in the previous chapter, suggested that it was possible to reliably obtain valid data from them. The GENEActiv activity monitor had previously been piloted and preferred to a competing device, as described in chapter 3, but the experience of using them within the

FaST-MP study split opinion. Three participants found no problem in using the devices and spoke about the devices positively, putting it on and forgetting about it. However, four participants spoke negatively about the GENEActive device, describing it as “too big”, “inappropriate for work”, “uncomfortable”, or generally finding the rubber strap uncomfortable. Mention was also made of the lack of watch face and unpleasant appearance; suggestions were made for the use of commercial devices, notably fitbits, which one of the participants wore normally, or apple watches. These had the benefit of being more functional and more pleasant in appearance.

Finally, criticism was also aimed at the facilities used for the modified shuttle walk tests. These occurred in the clinical trials unit within the Norfolk and Norwich University Hospital, itself a repurposed formed ward area. Whilst there was space for the necessary 9 metre track plus turning area, in practice it was surrounded by potential obstacles and could be crossed by members of staff within the unit accessing storage areas. Incidents had occurred where collisions or interrupted tests had resulted from other staff members walking onto the track by accident. A number of participants raised this during discussions:

“…when you do that [walking test], that needs a more defined area. Doing it in the corner of a corridor with filing cabinets, desks, chairs…” (006, Focus group 1)

“It wasn’t very good when I knocked that person walking was it?” (003, Focus group 1)

“I had a nurse come in at the last, she stopped me and I was in the peak wasn’t I? The peak of my physical fitness and she came across.” (002, Focus group 1)

“[It’s] just embarrassing when you’re walking down the corridor and someone comes around the corner.” (008, Focus group 2)

The majority of complaints came from members of focus group 1, which relates to problems with staff awareness early in the trial. This appeared to become less frequent as the trial progressed and staff in the unit were more aware of the test being conducted. Participants expressed frustration at these incidents occurring, reflecting the importance of obtaining the best possible result in their walking test for them and the failure to do this when they were interrupted, or embarrassment at running into a member of hospital staff. For some it was a humorous event which they laughed at when recollecting the event, but it was universally agreed that the location was not ideal for a modified shuttle walk test. One participant in focus group 3 went on to suggest a static test, considering a treadmill-based test to be preferable given the lack of space for a shuttle walk test to occur whilst

simultaneously allowing for incremental effort through faster speeds or increased inclines. This was also raised by another participant in another focus group who had noted that his exercise performance on the flat was good and may not reflect his true limitation:

"I mean, I done quite well with the walking test on the flat, but I think steps or something of that nature would give a bigger indication of how I struggle in my day to day." (008, Focus group 2)

An ergometer, be it treadmill or cycle-based, would have overcome these shortcomings but would rely upon appropriate equipment being available for this test as well as a pre-agreed protocol. If these steps were undertaken then it would appear that such static exercise equipment would be preferable to modified shuttle walk tests or other dynamic exercise tests. These tests require specific suitable locations, ideally a sports hall-type space, to perform safely. It is unlikely that many locations would have the facilities to do this within a hospital environment.

Discussion

During discussion of the potential changes that could be made to any related future studies a number of positive suggestions were made. The importance of ensuring an appropriate facility for conducting all outcome measures, specifically exercise-based ones such as the modified shuttle walk test, was reinforced through discussions with participants. Suggestions included:

- Future measures of exercise capacity should be considered in light of the expected localities where trial activities would expect to be taken place.
- The use of static equipment such as exercise bikes or treadmills may provide a reasonable alternative as many hospitals have access to a physiology laboratory for cardiopulmonary exercise testing, which could be utilised for this purpose.

The other major changes suggested related to the questionnaires and the activity monitors. Problems with both of them, notably the inability of the questionnaires to measure daily fluctuations in fatigue and the discomfort of wearing the activity monitors, led to the suggestion of a unified solution in the form of smart phones or smart watches – these devices would have a number of advantages:

- They are able to track activity levels as part of in-built health data collection

- They would be able to administer a simple questionnaire on a daily basis (e.g. rating fatigue level between 0-100), tracking daily fatigue levels.
- Different types of wrist straps are available for most smart watches, which would provide participants with the option to choose a strap that would be most comfortable for them.

Using the example of the apple watch, specific developer tools are available for health research; development of a program ('app') to collect both activity and fatigue data was considered an interesting solution by some participants and could be considered as a way of collecting the necessary resolution of data for any future study.

There were limitations to this study. Three focus groups were convened, allowing 14 of the 22 *FaST-MP* participants to discuss their views. However, new ideas and discussions were still being raised in the third focus group, suggesting that saturation was not reached within the three groups. There was also limited scope to explore in depth the impact of fatigue on daily living which, though not the primary rationale for undertaking focus group discussions, would have provided interesting data in a disease group that has not previously been investigated previously using these methods. Focus groups were not the ideal forum for discussing this in depth and contributed to the decision not to explore the impact of fatigue on individual patients prior to study entry. Future work would require semi-structured interviews with individuals to validate the findings relating to impact of fatigue on these patients. Finally, the focus groups occurred whilst *FaST*-MP was ongoing; as a result, participants were still blinded to their allocation at the time of the focus groups, so no insight could be gained into why participants believed such a significant benefit was possible on the placebo medication, or their reaction to finding out their allocation. Given the aims of the focus groups it was felt appropriate to explore these issues before unblinding, as well as potential difficulties with recalling trial experience for early trial participants if focus groups were undertaken after trial completion. Despite these limitations, the data collected here provided clear examples of benefit from neurostimulant therapy to individual patients, as well as identifying weakness of *FaST-MP*'s trial design and suggesting potential changes for any follow-up study.

References:

1. Braun V, Clarke V. Using thematic analysis in psychology. Qualitative Research in Psychology. 2006;3(2):77-101.
