# Supplementary material for: Feasibility of investigating methylphenidate for the treatment of sarcoidosis-associated fatigue (the FaST-MP study): a double-blind, parallel-arm randomised feasibility trial
Source: BMJ Open Respir Res. 2021 May 21;8(1):e000814. doi: 10.1136/bmjresp-2020-000814 (PMC8144057; doi:10.1136/bmjresp-2020-000814)
Supplement: Supplementary data [file bmjresp-2020-000814supp002.pdf]

Supplemental File S2 – Outcome measures

Tables:

Table 1 - Characteristics of non-fatigued, fatigued but ineligible, fatigued but declined participation and eligible randomised participants 2
Table 2 – Completion rates for questionnaires and other outcomes performed during the study 3
Table 3 – Activity monitor data relating to device return rates and data validity 4
Table 4 – Exit questionnaire responses by treatment allocation 4
Table 5 – Adverse event rates by treatment allocation; number of participants in each arm developing at least one AE within each individual organ system 4
Table 6 - Fatigue scores measured by Fatigue Assessment Scale (FAS) questionnaire during follow-up, including between-group differences 5
Table 7 - Fatigue scores measured by Functional Assessment of Chronic Illness Therapy - Fatigue (FACIT-Fatigue) questionnaire during follow-up, including between-group differences 5
Table 8 - Anxiety symptoms measured by the Hospital Anxiety and Depression Scale – Anxiety (HADS-A) score during follow-up, including between-group differences 6
Table 9 - Depression symptoms measured by the Hospital Anxiety and Depression Scale – Depression (HADS-D) score during follow-up, including between-group differences 6
Table 10 - Disease-related health status (general health status, lung and composite subscales) measured by the Kings Sarcoidosis Questionnaire (KSQ) during follow-up, including between-group differences 7

Figures:

Figure 1 - Change in weight (kg) across the duration of the study, stratified by treatment arm. Results are mean values with 95% confidence intervals 8
Figure 2 - Change in systolic BP (A), diastolic BP (B) and pulse rate (C) compared with baseline values across the duration of the study, stratified by treatment arm. Results are mean values with 95% confidence intervals 9

Table 1 - *Characteristics of non-fatigued, fatigued but ineligible, fatigued but declined participation and eligible randomised participants*

	**Group 1 – Non-fatigued**	**Group 2 – Fatigued, Ineligible**	**Group 3 – Fatigued, Eligible, Declined**	**Group 4 – Fatigued, Eligible, Randomised**
**Number (% total cohort)**	216 (56.1)	114 (29.6)	33 (8.6)	22 (5.7)*
**Age**	56.8 (14.6)	54.9 (13.5)	**49.3 (12.5)**	56.0 (9.2)
**Female sex (%)**	91 (42.1)	50 (43.8)	**20 (60.6)**	9 (40.9)
**Caucasian (%)**	210 (97.7)	113 (99.1)	30 (90.9)	22 (100)
**BMI**	32.8 (8.1)	32.6 (7.9)	30.4 (7.7)	31.5 (5.8)
**FEV1 (% predicted)**	91.5 (23.0)	**84.2 (22.7)**	92.6 (14.7)	92.7 (23.7)
**FVC (% predicted)**	99.8 (20.6)	**91.5 (21.0)**	98.1 (16.2)	100.0 (21.9)
**DLCO (% predicted)**	77.9 (22.4)	72.7 (19.3)	84.1 (9.3)	74.1 (20.0)
**KCO (% predicted)**	90.6 (23.7)	90.3 (18.8)	97.5 (12.8)	101 (11.3)
**Disease duration (years)**	9.1 (8.8)	7.4 (9.6)	**4.7 (4.6)**	7.8 (7.1)
**Pulmonary disease (%)**	212 (98.1)	113 (99.1)	32 (100)	22 (100)
**Extrapulmonary disease**	65 (30.1)	47 (41.2)	9 (27.3)	9 (40.9)
**Initial CXR stage 0/1/2/3/4 (%)**	22/85/38/45/10 (11/42.5/19/22.5/5)	15/42/20/24/5 (14.2/39.6/18.9/22.6/4.7)	3/16/5/5/0 (10.3/55.2/17.2/17.2/0)	4/10/2/1/1 (22.2/55.6/11.1/5.6/5.6)
**sACE (presentation)**	67.6 (42.0)	**84.7 (66.4)**	61.4 (28.7)	50.4 (27.6)
**sACE (latest)**	55.2 (31.2)	58.7 (40.8)	52.4 (20.1)	42.5 (19.3)
**Calcium (presentation)**	2.43 (0.19)	**2.49 (0.29)**	2.45 (0.15)	2.43 (0.10)
**Calcium (latest)**	2.41 (0.10)	2.42 (0.10)	2.39 (0.08)	2.43 (0.09)
**Receiving steroids (%)**	44 (20.4)	**63 (55.2)**	**3 (9.1)**	5 (22.7)
- **Mean dose (mg)**	- 7.4 (4.3)	- **15.4 (9.1)**	- **15.7 (12.5)**	- 10.3 (6.9)
**Receiving other immunosuppressant (%)**	**15 (6.9)**	19 (16.7)	5 (15.2)	3 (13.6)

Abbreviations: BMI – Body Mass Index; FEV1 – Forced Expiratory Volume in 1 second; FVC – Forced Vital Capacity; DLCO – Diffusing capacity of the lungs for carbon monoxide (single-breath hold); KCO – Transfer co-efficient of carbon monoxide; CXR – Chest X-Ray; sACE – Serum Angiotensin Converting Enzyme

*23 participants were randomised but one participant was discovered to be ineligible prior to receiving the intervention; their details are included in Group 2.

***Table 2** – Completion rates for questionnaires and other outcomes performed during the study*

Outcome	Expected data points - n	Missing Data points - n (%)
**FAS**	165	2 (1.2)
**FACIT-Fatigue**	165	2 (1.2)
**HADS**	121	4 (3.3)
**KSQ**	121	3 (2.5)
**EQ5D**	121	3 (2.5)
**SF36**	121	4 (3.3)
**Safety[1]**	104	5 (4.8)
**PSQI[2]**	43	2 (4.7)
**Spirometry (FEV1 and FVC)[3]**	60	3 (5.0)
**MSWT[4]**	60	7 (11.7)
**Activity monitor data[5]**	60	3 (5.0)
**Total**	1142	30 (2.6)

Acronyms: FAS – Fatigue Assessment Scale; FACIT-Fatigue – Functional Assessment of Chronic Illness Therapy – Fatigue; HADS – Hospital Anxiety and Depression Scale; KSQ – Kings Sarcoidosis Questionnaire; EQ5D – EuroQoL 5 Dimension 5 Level scale; SF36 – Short Form 36; PSQI – Pittsburgh Sleep Quality Index; FEV1 – Forced Expiratory Volume in 1 second; MSWT – Modified shuttle walk test

[1]Safety questionnaire was administered up to week 12; participants completing a truncated time period who completed study medications at week 12 did not all receive safety questionnaires at their final visit (4 out of 5 missing data points).

[2]PSQI only administered following major amendment approved in April 2017; expected data points refers to the number of visits where the questionnaire should have been administered after the study amendment was approved.

[3]All missing spirometry values occurred in a single participant who was unable to perform the test without suffering syncope; spirometry was not performed for this participant

[4]Six of the seven missing MSWT values occurred due to loss of facilities to undertake the test

[5]Missing data points for activity watches refers to an unreturned device (1 missing data point) or device not worn during wear period (2 missing data points).

Table 3 *– Activity monitor data relating to device return rates and data validity*

Period	No. participants	Devices returned (%)	Any data returned (%)	Minimum valid data[1] (%)	Wear for 24h periods[2]
Week 0	22	22 (100.0)	20 (90.9)	20 (90.9)	19 *(86.3)*
Week 12	22	21 (95.5)	20 (90.9)	19 (86.4)	17 *(77.3)*
Week 24	16	16 (100.0)	15 (93.8)	15 (93.8)	13 *(81.3)*
**Total**	60	59 (98.3)	55 (91.7)	54 (90.0)	49 *(81.6)*

[1]Defined as at least 10 hours of wear time per 24 hours in at least two weekdays and two weekend days

[2]The number of participants returning minimum valid data who wore the device for 24-hour periods on at least four days of the wear period

Table 4 *– Exit questionnaire responses by treatment allocation*

Exit Question	Methylphenidate (n=12)	Placebo (n=7)
If you were given the choice to continue receiving the study medication, would you want to?	11 (91.7)	5 (71.4)
Did you find participation in the study useful?	12 (100)	7 (100)
Given the chance again, would you still have taken part in this study?	12 (100)	7 (100)
Would you recommend taking part in any future study investigating methylphenidate to other patients?	12 (100)	7 (100)

Table 5 *– Adverse event rates by treatment allocation; number of participants in each arm developing at least one AE within each individual organ system*

CTCAE System Class	**Methylphenidate** Number of participants with ≥1 event (%)	**Placebo** Number of participants with ≥1 event (%)
Ear and labyrinth	2 (13.3)	0
Eye	1 (6.7)	3 (42.9)
Gastrointestinal	7 (46.7)	1 (14.3)
General disorders	2 (13.3)	2 (28.6)
Infections and infestations	1 (6.7)	1 (14.3)
Investigations	2(13.3)	0
Metabolism and nutrition	1 (6.7)	0
Musculoskeletal	5 (33.3)	1 (14.3)
Nervous system	10 (66.7)	3 (42.9)
Psychiatric	5 (33.3)	3 (42.9)
Respiratory	7 (46.7)	6 (85.7)
Reproductive system and breast	1 (6.7)	0
Skin and subcutaneous tissue	4 (26.7)	1 (14.3)
Vascular disorders	2 (13.3)	0
**Any**	14 (93.3)	7 (100.0)

Abbreviations: CTCAE – Common Terminology Criteria for Adverse Events

Table 6 *- Fatigue scores measured by Fatigue Assessment Scale (FAS) questionnaire during follow-up, including between-group differences*

	Methylphenidate		Placebo		Unadjusted difference[1,2]		Adjusted difference[1,3]	
**Week**	**n**	**Mean (S.D.)**	**n**	**Mean (S.D.)**	**Mean**	**95% C.I.**	**Mean**	**95% C.I.**
0	15	35.9 (7.7)	7	35.9 (8.8)	0.0	-7.7, 7.6	-	-
6	15	29.4 (11.5)	7	24.4 (9.2)	5.0	-5.4, 15.4	4.6	-2.9, 12.0
12	15	29.1 (9.4)	7	27.1 (12.9)	1.9	-8.2, 12.0	1.7	-5.4, 8.7
18	11	28.3 (9.3)	6	23.2 (7.0)	5.1	-4.2, 14.4	4.5	-3.1, 12.0
24	10	28.2 (8.7)	6	22.0 (8.6)	6.2	-3.4, 15.8	6.2	-1.4, 13.7
30	13	32.7 (9.5)	7	27.6 (12.1)	5.1	-5.2, 15.4	7.0	1.3, 12.8

Higher values indicate greater reported fatigue symptoms.

[1]Mean difference is equivalent to the *methylphenidate group value* minus the *placebo group value*. Positive values indicate the methylphenidate group value was higher than the placebo group, negative values indicate the methylphenidate group value was lower than the placebo group.

[2]Between-group difference tested using two-sample t-test with equal variance

[3]Between-group difference tested using linear regression analysis performed with adjustment for baseline fatigue severity and FAS score at visit 0

Table 7 *- Fatigue scores measured by Functional Assessment of Chronic Illness Therapy - Fatigue (FACIT-Fatigue) questionnaire during follow-up, including between-group differences*

	Methylphenidate		Placebo		Unadjusted difference[1,2]		Adjusted difference[1,3]	
**Week**	**n**	**Mean (S.D.)**	**n**	**Mean (S.D.)**	**Mean**	**95% C.I.**	**Mean**	**95% C.I.**
0	15	19.9 (11.0)	7	20 (10.8)	-0.1	-10.5, 10.3	-	-
6	15	27.9 (15.3)	7	33.7 (13.2)	-5.9	-19.9, 8.2	-5.5	-15.3, 4.2
12	15	28.6 (13.1)	7	29.3 (17.6)	-0.7	-14.6, 13.2	-0.4	-9.1, 8.2
18	11	30.2 (13.3)	6	36.0 (12.6)	-5.8	-19.9, 8.3	-4.5	-14.9, 5.9
24	10	29.0 (10.7)	6	39.2 (11.8)	-10.2	-22.5, 2.1	-9.2	-17.8, -0.6
30	13	23.9 (11.8)	7	28.3 (18.1)	-4.4	-18.8, 9.6	-6.9	-14.7, 1.0

Lower values indicate greater reported fatigue symptoms.

[1]Mean difference is equivalent to the *methylphenidate group value* minus the *placebo group value*. Positive values indicate the methylphenidate group value was higher than the placebo group, negative values indicate the methylphenidate group value was lower than the placebo group.

[2]Between-group difference tested using two-sample t-test with equal variance

[3]Between-group difference tested using linear regression analysis performed with adjustment for baseline fatigue severity and FACIT-Fatigue score at visit 0

Table 8 *- Anxiety symptoms measured by the Hospital Anxiety and Depression Scale – Anxiety (HADS-A) score during follow-up, including between-group differences*

	Methylphenidate		Placebo		Unadjusted difference[1,2]		Adjusted difference[1,3]	
**Week**	**n**	**Mean (S.D.)**	**n**	**Mean (S.D.)**	**Mean**	**95% C.I.**	**Mean**	**95% C.I.**
0	15	7.8 (3.3)	7	8.0 (4.9)	-0.2	-3.9, 3.5	-	-
6	14	7.1 (5.1)	6	4.0 (3.0)	3.1	-1.6, 7.9	1.9	-1.6, 5.3
12	15	6.8 (3.9)	7	4.3 (4.3)	2.5	-1.2, 6.3	2.7	0.4, 5.0
18	11	7.1 (3.6)	6	2.7 (2.7)	4.4	0.8, 8.1	4.3	1.8, 6.7
24	10	5.6 (1.8)	6	2.2 (2.7)	3.4	1.0, 5.8	3.4	1.6, 5.3
30	13	6.9 (3.7)	7	2.5 (2.5)	4.2	0.6, 7.8	3.6	0.6, 6.5

Higher values indicate greater reported anxiety symptoms.

[1]Mean difference is equivalent to the *methylphenidate group value* minus the *placebo group value*. Positive values indicate the methylphenidate group value was higher than the placebo group, negative values indicate the methylphenidate group value was lower than the placebo group.

[2]Between-group difference tested using two-sample t-test with equal variance

[3]Between-group difference tested using linear regression analysis performed with adjustment for baseline fatigue severity and HADS-A score at visit 0

Table 9 *- Depression symptoms measured by the Hospital Anxiety and Depression Scale – Depression (HADS-D) score during follow-up, including between-group differences*

	Methylphenidate		Placebo		Unadjusted difference[1,2]		Adjusted difference[1,3]	
**Week**	**n**	**Mean (S.D.)**	**n**	**Mean (S.D.)**	**Mean**	**95% C.I.**	**Mean**	**95% C.I.**
0	15	7.9 (3.0)	7	6.6 (4.5)	1.3	-2.0, 4.6	-	-
6	14	6.4 (5.2)	6	4.8 (3.6)	1.6	-3.4, 6.6	0.8	-3.4, 5.0
12	15	6.9 (4.3)	7	7.6 (6.6)	-0.7	-5.6, 4.2	-1.1	-5.5, 3.3
18	11	6.9 (3.1)	6	3.8 (2.1)	3.1	-0.1, 6.2	3.2	0.5, 5.9
24	10	6.4 (2.5)	6	3.2 (5.0)	3.2	-0.8, 7.2	2.7	-1.3, 6.7
30	13	8.1 (3.8)	7	3.8 (4.6)	4.2	-0.1, 8.4	4.3	1.5, 7.1

Higher values indicate greater reported symptoms of depression.

[1]Mean difference is equivalent to the *methylphenidate group value* minus the *placebo group value*. Positive values indicate the methylphenidate group value was higher than the placebo group, negative values indicate the methylphenidate group value was lower than the placebo group.

[2]Between-group difference tested using two-sample t-test with equal variance

[3]Between-group difference tested using linear regression analysis performed with adjustment for baseline fatigue severity and HADS-D score at visit 0

Table 10 *- Disease-related health status (general health status, lung and composite subscales) measured by the Kings Sarcoidosis Questionnaire (KSQ) during follow-up, including between-group differences*

		Methylphenidate		Placebo		Unadjusted difference[1,2]		Adjusted difference[1,3]	
	**Week**	**n**	**Mean (S.D.)**	**n**	**Mean (S.D.)**	**Mean**	**95% C.I.**	**Mean**	**95% C.I.**
**General Health State**	0	15	48.8 (12.8)	7	47.1 (11.8)	1.7	-10.2, 13.6	-	-
	6	14	58.5 (20.7)	7	59.4 (16.5)	-0.9	-19.7, 17.9	-4.0	-13.9, 6.0
	12	15	55.8 (14.7)	7	64.0 (26.2)	-8.3	-26.3, 9.8	-10.3	-21,7, 1.1
	18	11	58.9 (12.7)	6	68.6 (15.6)	-9.8	-24.6, 5.1	-9.7	-19.1, -0.2
	24	10	59.0 (14.4)	6	71.2 (13.1)	-12.3	-27.7, 3.2	-14.9	-23.7, -6.2
	30	13	53.9 (13.1)	7	57.1 (28.8)	-3.2	-22.7, 16.2	-8.8	-20.6, 3.1
**Lung**	0	15	58.8 (16.5)	7	43.7 (15.8)	15.1	-0.4, 30.7	-	-
	6	14	64.3 (19.8)	7	52.2 (9.1)	12.1	-4.5, 28.7	0.3	-11.7, 12.3
	12	15	58.4 (18.0)	7	53.7 (30.5)	4.7	-16.8, 26.2	-11.5	-26.7, 3.7
	18	11	59.4 (16.0)	6	67.6 (16.9)	-8.2	-25.8, 9.5	-11.7	-26.0, 2.6
	24	10	64.5 (20.8)	6	70.8 (15.9)	-6.3	-27.5, 15.0	-16.7	-33.7, 0.2
	30	13	64.0 (24.1)	7	61.8 (24.0)	2.3	-21.5, 26.0	-11.9	-31.6, 7.8
**Composite**	0	15	54.2 (7.7)	7	50.0 (6.7)	4.2	-2.8, 11.3	-	-
	6	14	59.7 (11.7)	7	55.9 (8.2)	3.8	-6.6, 14.2	-2.4	-6.9, 2.2
	12	15	55.8 (8.6)	7	61.0 (21.0)	-5.2	-18.1, 7.7	-11.1	-20.0, -2.2
	18	11	57.4 (7.6)	6	64.1 (12.4)	-6.6	-16.9, 3.6	-8.6	-15.1, -2.1
	24	10	59.5 (11.9)	6	66.3 (10.6)	-6.8	-19.5, 6.0	-12.4	-20.6, -4.2
	30	13	56.4 (10.5)	7	59.3 (19.1)	-2.8	-16.6, 10.9	-9.6	-18.0, -1.2

Higher values indicate greater health-related quality of life and lower burden of disease-related symptoms.

[1]Mean difference is equivalent to the *methylphenidate group value* minus the *placebo group value*. Positive values indicate the methylphenidate group value was higher than the placebo group, negative values indicate the methylphenidate group value was lower than the placebo group.

[2]Between-group difference tested using two-sample t-test with equal variance

[3]Between-group difference tested using linear regression analysis performed with adjustment for baseline fatigue severity and KSQ subscale scores at visit 0

Figure 1 *- Change in weight (kg) across the duration of the study, stratified by treatment arm. Results are mean values with 95% confidence intervals*

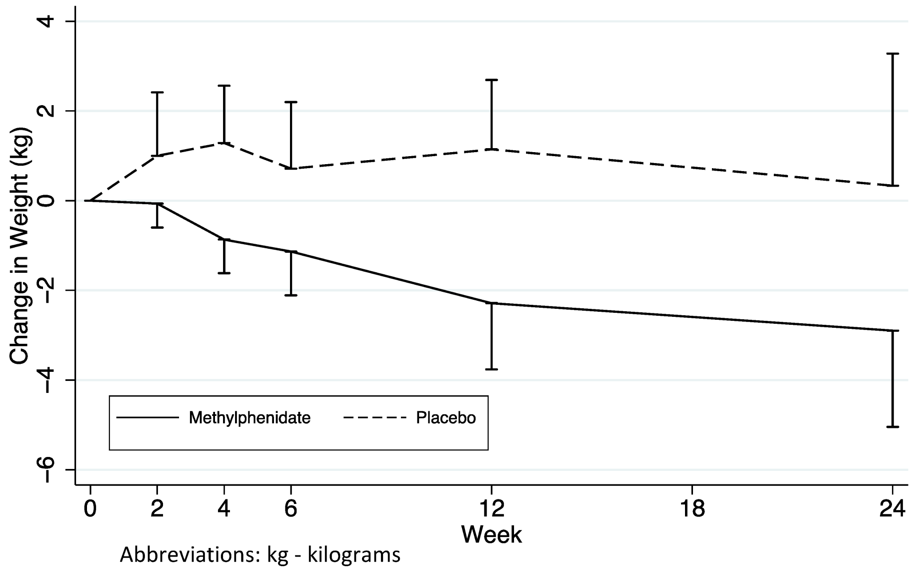

Abbreviations: kg - kilograms
All participants provided weight recordings at week 0, 2, 4, 6, 12 and 24.
Values for each visit after week 0 denote change from baseline value

Figure 2 *- Change in systolic BP (A), diastolic BP (B) and pulse rate (C) compared with baseline values across the duration of the study, stratified by treatment arm. Results are mean values with 95% confidence intervals*

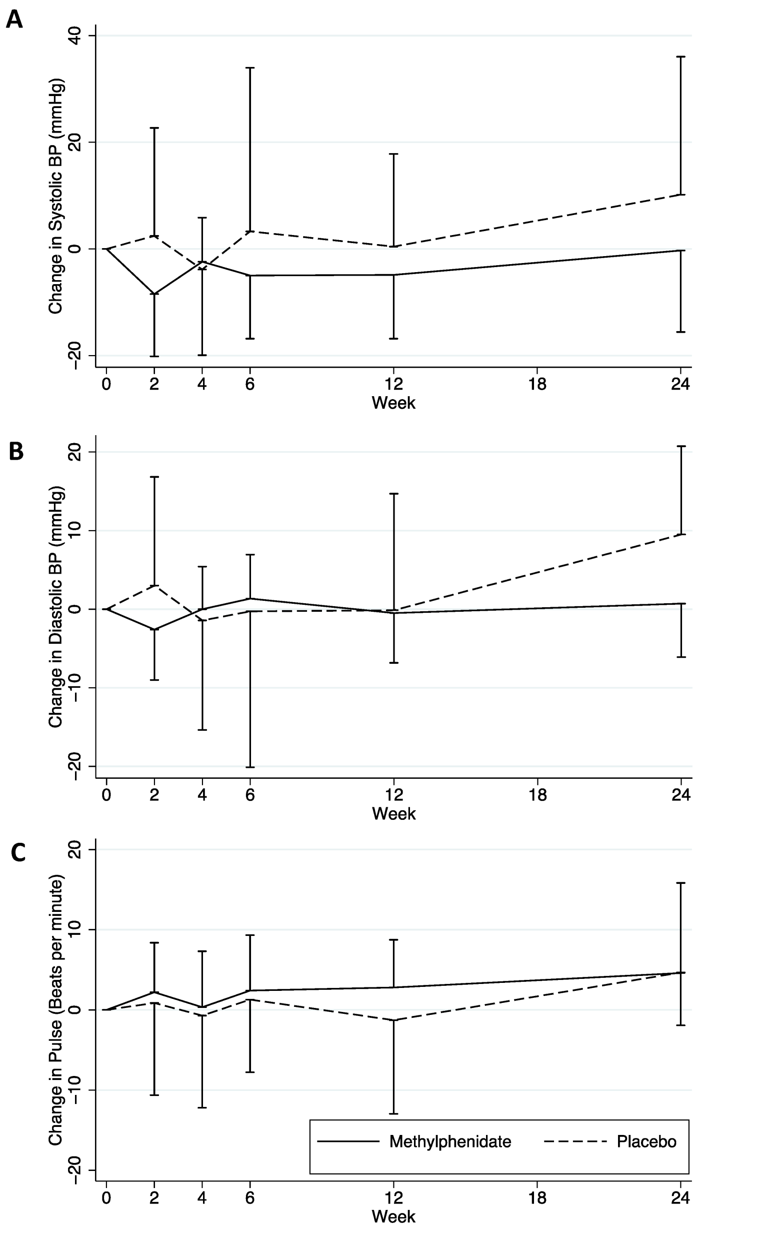

Acronyms: BP – Blood pressure; mmHg – millimetres of mercury
All participants provided systolic BP, diastolic BP and pulse recordings at week 0, 2, 4, 6, 12 and 24.
Values for each visit after week 0 denote change from baseline value
